# Contemporary and Novel Imaging Studies for the Evaluation of Erectile Dysfunction

**DOI:** 10.3390/medsci7080087

**Published:** 2019-08-09

**Authors:** Eric Chung

**Affiliations:** 1 AndroUrology Centre, Brisbane, QLD 4000, Australia; ericchg@hotmail.com; Tel.: +617-3832-1168; 2 Princess Alexandra Hospital, University of Queensland, Brisbane, QLD 4102, Australia; 3 Macquarie University Hospital, Sydney, NSW 2109, Australia

**Keywords:** erectile dysfunction, imaging, colour duplex ultrasound, angiography, MRI, PET, EEG, cavernosography, cavernosometry

## Abstract

Traditionally, it was thought that the pathogenesis of erectile dysfunction (ED) can be divided into psychological and organic factors. However, recent literature supports the development and progression of ED due to multidimensional alterations of a complex interplay of central and peripheral systems, from neural cognitive and efferent networks to loco-regional neuro-hormonal factors which are responsible for impaired penile vascular hemodynamics and ensuing lack of, or suboptimal, blood flow into the penis and/or veno-occlusive dysfunction. It is recognised that ED is strongly correlated with cardiovascular health and published clinical guidelines advocate screening for cardiovascular and metabolic risk factors in men presenting with ED. Over the past few decades, various imaging modalities have been developed and utilised to provide objective evaluation for ED to better characterise the state of penile health and exclude psychogenic components. The following article evaluates current and emerging imaging diagnostic tools for ED.

## 1. Introduction

Erectile dysfunction (ED) is defined as the persistent inability to attain and/or maintain an erection sufficient for sexual intercourse [1]. The pathogenesis of ED is attributed to both psychogenic factors, as well as physiological alterations in neural, vascular, hormonal and endothelial functions. It is accepted that development and progression of ED involve a complex interplay of central and peripheral systems, from neural cognitive and efferent networks (processing and integration of tactile, visual, olfactory, and imaginative stimuli) [2] to loco-regional neuro-hormonal factors are responsible for penile vascular hemodynamics and ensuing erection responses in a healthy penis.

Published literature supports the association between ED and cardiovascular health, with underlying endothelial dysfunction plays a pivotal role. The modifiable risk factors for cardiovascular disease are shared with ED such as hypertension, hyperlipidemia, diabetes, central obesity, lack of physical exercise, cigarette smoking and poor diet. Of clinical importance, is the recognition that ED is an independent marker of increased risk and overall health. The introduction of oral phosphodiesterase type 5 inhibitor has transformed the way clinicians approach men with ED and perhaps avoids the need for further specialised testing apart from screening these men for cardiovascular and metabolic risk factors. Nonetheless, specialised imaging studies can provide a further anatomical and functional evaluation of the underlying cavernosal arterial inflow, veno-occlusive mechanism and smooth muscle status. The following article evaluates current and emerging imaging diagnostic tools for ED.

## 2. Current (Traditional) Imaging Tests

### 2.1. Penile Colour Doppler/Duplex Ultrasound with Intracavernosal Vasoactive Injection Test

Penile colour duplex ultrasound (CDU) provides a relatively inexpensive, simple and safe assessment for various penile ultra-structures such as cavernosal smooth muscle, tunical plaque and vascular parameters [3]. Penile CDU is usually performed with concurrent use of intracavernosal vasoactive drug(s) injection to provide adequate penile erection at the time of imaging study.

Clinical parameters such as cavernous arterial diameter, the direction of blood flow, peak systolic velocity (PSV), acceleration time, end diastolic velocity (EDV) and resistance index provides a measurement of the underlying penile hemodynamics [4]. The normal value for post-injection cavernosal arterial flow is PSV greater than 25 to 35 cm per second (cm/s), while the normal value for EDV of less than 5 cm/s excludes possible veno-occlusive dysfunction or venous leak. In men who do not achieve complete penile tumescence during penile CDU, flaccid penile acceleration has been proposed as an alternative CDU parameter since it is an indirect measurement of vascular stiffness [5]. In addition, penile CDU allows for further characterization of the cavernosal smooth muscle state such as the presence of intracavernosal fibrosis and calcification, septal scar and tunical disease [3,6]. Published studies correlating post-occlusive vasodilation of cavernous arteries in men with ED may provide a functional assessment of cavernosal endothelium state [7], although controversies exist whether this finding is related to the direct effect of tissue ischemia or an increase in shear stress of the endothelial wall from occlusion of the vascular flow.

### 2.2. Cavernosometry and Cavernosography

Dynamic infusion cavenosography and cavernosometry (DICC) is considered the gold standard test to assess both arterial insufficiency and veno-occlusive dysfunction (venous leak) [8]. The main objective of cavernosometry is to record the relationship between corpus cavernosum infusion rate required to sustain an intracavernosal pressure that equals the mean arterial systemic blood pressure, while cavernosography identifies the specific location of cavernous venous leak [9].

While DICC is thought to be more accurate that penile CDU in diagnosing venous leak, it is time-consuming and is an invasive test that requires clinical expertise to interpret the radiographic findings [10]. In most cases, repeated intracavernous injections of vasoactive agents are often necessary, and that it is often difficult to obtain accurate measurement with complete corporal smooth muscle relaxation. Since venous ligation surgery has been largely discontinued due to poor long-term outcome, the clinical utility of DICC has diminished.

### 2.3. Penile Angiography

Penile angiography is often regarded as the gold standard in the diagnosis of arteriogenic ED [11], and digital subtraction angiography (DSA) is performed to assess significant vascular anatomical variations or stenosis after intracavernosal injection of a vasoactive agent. Penile angiography is mandatory for men who are candidates for penile revascularization surgery.

Studies have shown a strong correlation between internal pudendal artery stenosis and the presence of angiographic coronary artery disease in men with ED [12] and the presence of a specific internal pudendal stenotic lesion can predict the onset of ED in men [13]. However, arterial malformations or anatomical variations are often common [14], and the acceptance of penile prosthesis implant as an effective and permanent solution for medically refractory ED has largely superseded penile revascularization surgery even in men with focal arteriogenic ED [15].

### 2.4. Nocturnal Penile Tumescence Test

Although a nocturnal penile tumescence test (NPT) does not technically qualify as an imaging test, it has been utilised extensively in the past to diagnose men with psychogenic ED [16]. The RigiScan device (Gotop Medical Inc., St. Paul, MN, USA) is an ambulatory machine that measures the frequency, duration and rigidity of nocturnal and provocative penile erections, and provides a more accurate assessment of penile rigidity, compared to previous rudimentary contraptions such as stamps, snap gauge band and erectiometer.

At present, there is a lack of consensus on acceptable NPT parameters to define true ED and many confounding variables can play a role, resulting in non-discrimination of the aetiologies [17]. The duration and intensity of nocturnal erections are likely related to the patient’s age, the environment of NPT study and mental state of the patient on the day of the study [18].

## 3. Newer and Novel Imaging Tests

### 3.1. CT Angiography and MR Angiography

Technological advances in the past decade have allowed for higher resolution and better definition of segmental vessels, including those of pudendal and penile arterial lesions in the evaluation of men with vascular ED. Computed tomography angiography (CTA) is a relatively inexpensive and minimally invasive imaging modality that has reasonable accuracy in localising internal pudendal arterial stenosis when compared to DSA [19]. More recently, magnetic resonance angiography (MRA) has replaced CTA as the imaging modality of choice due to better image resolution and a more accurate angiography method in the work-up of arteriogenic ED [20].

Recent literature found that high-resolution MRI of the penis can accurately predict underlying smooth muscle viability in patients presenting with priapism, with close correlation to that of corporal cavernosal biopsy [21]. Another study showed strong correlation and regression analyses between the T2 isotropic sequence on the MRI and erectile function scores [22], and MRA can provide a precise angio-anatomic evaluation of segmental arteries destined to the penis to assist surgeons to preserve them and reduce the risk of postoperative ED [23].

### 3.2. PET Scan

Existing anatomical imaging modalities such as penile CDU, CT or MRI provide a useful assessment of cavernous smooth muscle and blood flow parameters. Unlike their anatomic counterparts, molecular imaging modalities are capable of detecting microscopic processes such as inflammation and microcalcification, which may precede ultrastructural changes in ED development and progression.

The ^18^F-fluorodeoxyglucose (^18^F-FDG) positron emission tomography (PET) is the most widely utilized PET radiotracer by far, and was among the first molecular probes used for the assessment of atherosclerosis. Early studies showed a correlation between ^18^F-FDG activity within the vasculature and atherosclerotic and cardiovascular risk factors, thereby promoting its utility as a useful diagnostic agent [24]. By contrast, the ^18^F-sodium fluoride (^18^F-NaF) is a specific marker of bone mineralization that has traditionally been used in diagnosing metastatic bone cancer, but has recently been applied to vascular calcification and has shown considerable promise in allowing the evaluation of patients at risk of atherosclerosis [25]. Combined PET/CT imaging of atherosclerosis using ^18^F-NaF has the potential to identify pathologically high-risk nascent microcalcification. While macrocalcification confers plaque stability, microcalcification is a key feature of high-risk atheroma and is associated with increased morbidity and mortality. It differs from ^18^F-FDG PET in its molecular binding characteristics and, thus, the manner in which it illustrates disease burden. Several studies have shown that vascular uptake of ^18^F-NaF is not only correlated with advancing age but also with risk factors for atherosclerotic and cardiovascular disease [26] and that NaF uptake in penile vessels suggests that atherosclerosis is associated with ED in prostate cancer patients [27].

### 3.3. Electroencephalogram and Functional MRI Neuro-Imaging Techniques

Various neuroimaging studies have identified critical changes in cerebral activity and structures within the brain regions during the processes of sexual arousal and penile erection, and this provides new insights into the complex relationships between the various pathophysiological mechanisms and the neuroanatomical processes responsible for ED [28]. Electroencephalogram (EEG) recordings of the human brain activity during different sexual responses show various changes of electrical activity across the different parts of the human brain.

Over the past decade, the development of higher spatial resolution neuro-imaging techniques, such as functional MRI (fMRI), has largely replaced surface EEG study [29]. Various neuroimaging studies had identified changes in cerebral activity and structures during the processes of sexual arousal [30]. Psychogenic ED showed alteration within the right superior frontal gyrus (dorsolateral), superior parietal gyrus, para-hippocampal gyrus and left temporal pole (superior temporal gyrus), which were thought to regulate emotional and cognitive processes [31].

Combining EEG neuro-imaging techniques with standard clinical and psychological approaches could provide major advances to improve the understanding of the human sexual function. However, there are considerable limitations in the capabilities of these methods to adequately estimate subcortical brain sources from EEG recordings [32]. While newer fMRI studies have been undertaken to identify the cortical networks associated with various sexual thoughts and functioning [33] and likely provide the insight for a global neuroanatomical model of cortical networks, this new investigative tool remains experimental and further translational neuroscience studies are required to study different distinct cerebral networks and validate the clinical findings.

## 4. Conclusions

Despite the dichotomy between organic and psychogenic ED, many clinicians would agree that most often, organic ED and psychogenic ED co-exist together. Nonetheless, with recent emphasis on the association between ED and endothelial dysfunction, it remains pivotal that the basic workup for men with ED allows for screening of cardiovascular risk factors. However, it is likely that the development and progression of ED is multifactorial in nature. Since no one can be completely certain to have excluded all possible organic aetiologies for ED, the exact diagnosis of ED cannot be of exclusion, but rather of probability.

The basic evaluation for men with ED includes routine blood tests such as complete blood count, serum chemistry, fasting glucose, lipid profile and serum total testosterone levels. Then, depending on clues raised during one’s history and physical exam, more directed lab-work could be conducted. While it is important to factor cost-analysis in the decision-making process, it is often difficult due to many confounding factors such as healthcare utilization, private reimbursement rate, perceptions and benefits of diagnostic tests, and referral patterns.

The increase in our understanding of the alterations in penile ultrastructure and neurohumoral mechanisms responsible for erectile physiology have resulted in the adoption of various imaging modalities to diagnose ED and predict likely effective treatment pathways.
